# Recreational Physical Activity and Premenstrual Syndrome in Young Adult Women: A Cross-Sectional Study

**DOI:** 10.1371/journal.pone.0169728

**Published:** 2017-01-12

**Authors:** Aimee R. Kroll-Desrosiers, Alayne G. Ronnenberg, Sofija E. Zagarins, Serena C. Houghton, Biki B. Takashima-Uebelhoer, Elizabeth R. Bertone-Johnson

**Affiliations:** 1 Department of Quantitative Health Sciences, University of Massachusetts Medical School, Worcester, MA, United States of America; 2 School of Public Health and Health Sciences, University of Massachusetts Amherst, Amherst, MA, United States of America; Victoria University, AUSTRALIA

## Abstract

**Introduction:**

It is estimated that up to 75% of premenopausal women experience at least one premenstrual symptom and 8–20% meet clinical criteria for premenstrual syndrome. Premenstrual syndrome substantially reduces quality of life for many women of reproductive age, with pharmaceutical treatments having limited efficacy and substantial side effects. Physical activity has been recommended as a method of reducing menstrual symptom severity. However, this recommendation is based on relatively little evidence, and the relationship between physical activity, premenstrual symptoms, and premenstrual syndrome remains unclear.

**Methods:**

We evaluated the relationship between physical activity and premenstrual syndrome and premenstrual symptoms among 414 women aged 18–31. Usual premenstrual symptom experience was assessed with a modified version of the Calendar of Premenstrual Experiences. Total, physical, and affective premenstrual symptom scores were calculated for all participants. Eighty women met criteria for moderate-to-severe premenstrual syndrome, while 89 met control criteria. Physical activity, along with dietary and lifestyle factors, was assessed by self-report.

**Results:**

Physical activity was not significantly associated with total, affective, or physical premenstrual symptom score. Compared to the women with the lowest activity, women in tertiles 2 and 3 of activity, classified as metabolic equivalent task hours, had prevalence odds ratios for premenstrual syndrome of 1.5 (95% CI: 0.6–3.7) and 0.9 (95% CI: 0.4–2.4), respectively (p-value for trend = 0.85).

**Conclusions:**

We found no association between physical activity and either premenstrual symptom scores or the prevalence of premenstrual syndrome.

## Introduction

It is estimated that up to 75% of premenopausal women experience at least physical or emotional symptom in the two weeks before menses begins [1,2]. Common premenstrual symptoms range from emotional and behavioral symptoms such as depression, angry outbursts, irritability, crying spells, anxiety, confusion, social withdrawal, poor concentration, sleep disturbance, and thirst and appetite changes, to physical symptoms including breast tenderness, bloating and weight gain, headache, swelling of the hands or feet, and aches or pains [1].

While premenstrual symptoms are common, a smaller proportion of women meet clinical criteria for premenstrual syndrome (PMS). Occurring in 8–20% of premenopausal women, PMS is associated with substantial reduction in quality of life [3,4]. The causes of PMS are unclear, though an underlying neurobiological vulnerability to normal fluctuations in the circulating sex hormones levels during the menstrual cycle [5,6] is thought to contribute. Hormonal fluctuations may also alter brain neurotransmitter or neuropeptide function [5,6], contributing to both emotional and physical symptoms. In particular, some studies have observed luteal phase abnormalities in serotonin function in women with PMS compared to symptom free controls [5]. Despite the high prevalence of PMS, there are relatively few treatment options available. Recreational physical activity has been recommended as a method of reducing PMS occurrence and severity and as an alternative to pharmaceutical treatments in easing symptoms [7]. Aerobic physical activity may increase endorphin levels [5,8], decrease levels of estradiol and other steroid hormones, [9,10] improve transport of oxygen in muscles [9], reduce cortisol levels [9], and improve psychological well-being [8,11]. It is hypothesized that frequent physical activity may, at least in part, reduce PMS and premenstrual symptoms through these mechanisms.

Previous observational studies examining the relationship between physical activity and PMS have demonstrated inconsistent findings. Several studies suggest a protective effect [8,11–19], one showed no association [20], and two studies found higher levels of premenstrual symptoms among women who exercised more compared to those exercising less or not at all [21,22]. Multiple factors could contribute to the differences in findings between studies. First, only one study [21] adjusted for body mass index (BMI), which has been positively associated with premenstrual symptoms and PMS [23,24] and is correlated with physical activity level. Failure to control for BMI may thus lead to confounding due to the underlying relationship between physical activity and BMI. Secondly, results from observational studies assessing premenstrual symptoms and physical activity may be affected by reverse causality, as women may use exercise as a way to relieve symptoms.

To address the limitations of existing studies, we explicitly evaluated the prevalence of premenstrual symptoms and PMS in 414 young adult women. As the etiology of PMS is still unclear, identifying modifiable risk factors is important to improve the quality of life for women of reproductive age [6]. Our objectives were to 1) examine the association between physical activity and prevalent PMS and premenstrual symptom severity; 2) investigate the impact of confounding by BMI; and 3) evaluate the potential for reverse causality through self-reported use of exercise to treat premenstrual symptoms. We hypothesized that young women who participated in recreational physical activity would be less likely to experience PMS and moderate-severe menstrual symptoms compared to women who participated in less physical activity; results adjusted for BMI would be stronger than those unadjusted for BMI; and including a self-reported “exercise to treat PMS” variable in our models would control for the potential of reverse causality.

## Methods

### Study design and population

This study was approved by the Institutional Review Board at the University of Massachusetts Amherst. All participants provided their written informed consent to participate. Study participants were healthy women aged 18–31 recruited into the UMass Vitamin D Status Study between 2006 and 2014. The study protocol has been described in detail previously [25,26]. In brief, women were ineligible to participate if they were pregnant or not currently menstruating; were experiencing untreated depression; reported high blood pressure or elevated cholesterol, kidney or liver disease, bone disease such as osteomalacia, digestive disorders, rheumatologic disease, multiple sclerosis, thyroid disease, hyperparathyroidism, cancer, type 1 or type 2 diabetes, or polycystic ovaries; or were currently using corticosteroids. All study measurements took place during the late luteal/high hormone phase of each participant’s menstrual cycle (*i*.*e*., 5–7 days before the anticipated start of menses). Participants reported the actual start date of next menses via email to confirm that measurements were conducted during the luteal phase for each participant.

### Assessment of physical activity

Participation in recreational physical activity was assessed by questionnaire, on which women were asked to report the average time they spent each week over the past month engaging in specific activities including walking, hiking, jogging, running, bicycling, aerobics or dancing, tennis or other racket sports, swimming, yoga or pilates, and weight training. Response options ranged from zero minutes to 11+ hours per week. Questionnaire responses were used to calculate metabolic equivalent task (MET) hours per week, with one MET defined as the energy expended while sitting quietly [27]. Our questionnaire was adapted from that used similarly in the Nurses’ Health Study 2; the validity of these questions to assess physical activity among young women has been demonstrated previously [28].

### Assessment of premenstrual symptoms and PMS

Information on premenstrual symptoms was collected through self-reported questionnaire using a modified version of the Calendar of Premenstrual Experiences (COPE) designed by Mortola *et al*. [29] and similar to that used in the Nurses’ Health Study 2 Premenstrual Syndrome Sub-Study [30]. In a recent validation study, we found that the sensitivity of the modified COPE questionnaire versus prospective symptom diaries to identify PMS cases was 73%, while positive predictive value was 80% and the specificity of our questionnaire versus daily symptom diaries to identify controls was 73%, while the negative predictive value was 100% [31].

Participants were asked if they experienced any of 26 different symptoms most months and to indicate the usual severity of each symptom as none, mild, moderate, or severe. Responses were individually scored from 1–4 (none = 1, mild = 2, moderate = 3, severe = 4) and summed across symptoms to derive subscores for affective symptoms (n = 8 symptoms; range of possible scores = 8–32), physical or behavioral symptoms (n = 18 symptoms; range of possible scores = 18–72), and a total premenstrual symptom score (range of possible scores = 26–104). Additionally, participants were asked to report whether they experienced relationship discord with family or a partner; relationship discord with friends or coworkers; poor work performance or attendance; or social isolation, and to indicate the severity of each as not a problem, mild, moderate, or severe. Women were also asked to report whether they had ever used exercise, dietary supplements or other factors to treat their symptoms.

A woman was considered a case if she reported moderate or severe symptoms starting at the luteal phase and ending within a week of the start of menses, following methods used in previous analyses in our study population [31,32]. Controls were women who reported no or mild symptoms with little impact on life activities and relationships. Both cases and controls had no evidence of a comorbid psychiatric disorder, including use of anti-depressant medications (see S1 Appendix for more detailed information on case/control identification).

### Covariate assessment

Covariate information was collected through questionnaire measuring factors including age, race, menstrual cycle information (including age at first menses, number of years after onset of menstruation before cycle became regular, current usual length of menstrual cycle, number of days of bleeding during period, and current usual pattern of menstrual cycles), current oral contraceptive use, pregnancy status (ever vs. never) and ever smoker status (defined as >20 packs of cigarettes smoked in lifetime). Two modified versions of the Harvard Food Frequency Questionnaire was used to estimate daily intake of vitamin D (IU), vitamin B6 (mg), and calcium (mg) consumption intake from both dietary and supplemental sources, as well as daily intake of alcohol (grams) and caffeine (mg) (Original version, 2006–08; Updated version, 2009–2014). Nutritional intake variables were adjusted for total energy using the residual method [33]. Height and weight were measured directly and used to calculate BMI (weight [kg]/height [m]^2^).

### Statistical analysis

We started by examining bivariate relationships of characteristics of our study participants by PMS case/control status. For continuous factors, Student’s t-test was used to examine mean differences between PMS cases and controls. Bivariate associations for categorical variables were examined using likelihood ratio chi-squared tests. To determine covariates in our multivariable models, we ran a forward selection logistic regression model (α = 0.10) predicting case/control status from tertiles of physical activity (in METs). Age and BMI were included as *a priori* covariates. Additional covariates in our multivariable models included calcium intake, age at first menses (≤12 vs. >12), and ever smoker (yes vs. no). Finally, to address the possibility of reverse causality, we took into consideration whether women reported specifically using exercise to treat premenstrual symptoms. We ran a complete case analysis in our multivariable models and utilized the Hosmer-Lemeshow test to examine goodness-of-fit in our logistic models.

To assess the association between physical activity and risk of PMS, we examined physical activity by continuous METs per week and by tertiles of physical activity. To examine if physical activity was associated with the severity of premenstrual symptoms overall, we evaluated how physical activity was associated with each of the three premenstrual symptom scores (total, affective, and physical) using linear regression. We included all women in our sample in this analysis to evaluate how physical activity correlates with symptoms across a wide range of symptom experience and severity.

The relationship between physical activity and PMS status was modeled using multivariable logistic regression to estimate odds ratios (ORs) and 95% confidence intervals (CIs). Two analyses were conducted; the first examined physical activity categorized into tertiles of METs per week and the second examined continuous physical activity in units of 10 METs per week. We included only women who met PMS case or control criteria in these models; women meeting neither criterion were excluded. This allowed us to compare women at extreme ends of the menstrual symptom experience and is consistent with previous studies of behavioral risks for PMS [25,30].

Data analyses were performed using SAS software, Version 9.4 for Windows (SAS Institute Inc., Cary, NC, USA).

## Results

A total of 414 women participated in our study. The average age of the participants was 21.0 years (+/- 2.6), mean BMI was 23.1 kg/m^2^ (+/- 3.4), and 45% of our sample currently used oral contraceptives (Table 1). Of these women, 80 (19.3% of our sample) met criteria for PMS, while 89 (21.5%) met control criteria. PMS cases were more likely to have experienced their first menses at age 12 or younger (p<0.01) compared to controls. Controls had a higher intake of calcium than cases (p<0.01). Other factors did not differ statistically between PMS cases and controls.

**Table 1:** Characteristics of study participants (n = 414) and comparison between PMS cases (n = 80) and controls (n = 89)*.

Characteristics^§^	Total Sample	PMS Case	PMS Control	p-value^†^
	n = 414	n = 80	n = 89	
	Mean ± SD	Mean ± SD	Mean ± SD	
Age (years)	21.0 ± 2.6	20.9 ± 2.5	20.9 ± 2.5	0.94
BMI (kg/m^2^)	23.1 ± 3.4	22.9 ± 3.3	22.7 ± 3.0	0.60
Vitamin D intake (IU/day)	132.1 ± 104.3	121.2 ± 96.8	151.2 ± 119.1	0.08
Calcium intake (mg/day)*	423.2 ± 164.7	385.6 ± 134.6	461.0 ± 185.4	<0.01
Vitamin B6 intake (mg/day)*	2.2 ± 3.6	2.2 ± 3.9	1.6 ± 1.3	0.25
Current alcohol intake (grams/day)*	2.7 ± 2.9	2.5 ± 2.0	2.7 ± 2.8	0.68
Daily caffeine consumption (mg/day)*	34.2 ± 35.4	37.5 ± 35.0	28.8 ± 36.5	0.16
	n (%)	n (%)	n (%)	
Race				
• White	340 (82.1)	59 (73.8)	75 (84.3)	0.09
• Other	74 (17.9)	21 (26.3)	14 (15.6)	
Age at first menses				
• ≤12	221 (53.4)	53 (66.3)	39 (43.8)	<0.01
• >12	193 (46.6)	27 (33.8)	50 (56.2)	
Number of years after onset of menses before cycle became regular				
• <1 year	132 (32.0)	21 (26.3)	30 (33.7)	0.70
• 1–2 years	138 (33.4)	27 (33.8)	34.8)	
• 3–4 years	85 (20.6)	21 (26.3)	20.2)	
• >5 years	30 (7.3)	5 (6.3)	(3.4)	
• Never	28 (6.8)	6 (7.5)	7 (7.9)	
Current usual length of menstrual cycle				
• <21 days	5 (1.2)	1 (1.3)	1 (1.1)	0.61
• 21–25 days	94 (22.7)	17 (21.3)	24.7)	
• 26–31 days	261 (63.0)	54 (67.5)	67.4)	
• 32–39 days	31 (7.5)	4 (5.0)	(3.4)	
• >40 days	12 (2.9)	2 (2.5)	(3.4)	
• Too irregular to calculate	11 (2.7)	2 (2.5)	0 (0.0)	
Days of bleeding during menses				
• ≤3 days	56 (13.6)	11 (13.8)	15 (16.9)	0.85
• 4–5 days	274 (66.3)	52 (65.0)	65.2)	
• 6–7 days	80 (19.4)	15 (18.9)	16.9)	
• ≥8 days	3 (0.7)	2 (2.5)	1 (1.1)	
Current usual pattern of menstrual cycles (days before or after expected menses)				
• Extremely regular (within 1–2 days)	157 (37.9)	25 (31.3)	38 (42.7)	0.37
• Very regular (within 3–4 days)	116 (28.0)	29 (36.3)	28.1)	
• Regular (within 5–7 days)	110 (26.6)	23 (28.8)	23.6)	
• Usually/always irregular	31 (7.5)	3 (3.8)	5 (5.6)	
Ever been pregnant				
• Yes	11 (2.7)	4 (5.0)	3 (3.4)	0.60
• No	403 (97.3)	76 (95.0)	86 (96.6)	
Current oral contraceptive use				
• Yes	186 (44.9)	42 (52.5)	49 (55.1)	0.74
• No	228 (55.1)	38 (47.5)	40 (44.9)	
Ever smoker				
• Yes	46 (11.1)	14 (17.5)	7 (7.9)	0.06
• No	368 (88.9)	66 (82.5)	82 (92.1)	

*Numbers/percentages may not sum to column total due to missing data; n = 348 (n = 76 controls and n = 61 cases) for calcium, vitamin B6, alcohol, and caffeine intake;

^§^Values are n (%) for categorical variables and mean ± standard deviation for continuous variables;

^†^P-values from Likelihood ratio χ^2^ test for categorical variables and Student’s t-test for continuous variables comparing PMS cases and controls.

PMS symptom severities are presented in Table 2. All 26 physical and affective symptoms experienced for at least several days prior to the onset of menses were reported to be more severe by cases compared to controls; cases reported an average total symptom severity score 23 points higher than controls (p < .01). The symptoms most frequently rated as “severe” by all participants included the tendency to cry easily (25.2%), emotional hypersensitivity (22.8%), irritability (22.1%), food cravings (20.5%), mood swings (17.3%), cramps (14.2%), and appetite changes (13.4%) (data not shown).

**Table 2:** Severity of premenstrual symptoms* of all study participants (n = 414) and of women meeting PMS case (n = 80) and control criteria (n = 89).

	Total Sample	PMS Case	PMS Control	p-value^§^
	n = 414	n = 80	n = 89	
	Mean ± SD	Mean ± SD	Mean ± SD	
*Physical Symptoms*				
• Abdominal bloating	2.2 ± 0.8	2.7 ± 0.7	1.6 ± 0.5	<0.01
• Abdominal cramping	2.3 ± 1.0	2.7 ± 1.1	1.5 ± 0.5	<0.01
• Acne	2.1 ± 0.8	2.2 ± 0.8	1.8 ± 0.7	0.04
• Back pain	1.9 ± 0.9	2.4 ± 1.1	1.4 ± 0.5	<0.01
• Breast tenderness	2.0 ± 1.0	2.4 ± 1.0	1.4 ± 0.5	<0.01
• Confusion	1.1 ± 0.4	1.2 ± 0.5	1.0 ± 0.0	0.01
• Diarrhea/constipation	1.7 ± 0.9	2.1 ± 1.0	1.2 ± 0.4	<0.01
• Dizziness	1.2 ± 0.5	1.3 ± 0.7	1.0 ± 0.0	<0.01
• Fatigue	2.1 ± 1.0	2.5 ± 0.9	1.2 ± 0.4	<0.01
• Food cravings	2.4 ± 1.1	3.0 ± 1.0	1.5 ± 0.7	<0.01
• Forgetfulness	1.2 ± 0.5	1.3 ± 0.5	1.0 ± 0.2	0.02
• Headache	1.7 ± 0.9	1.9 ± 1.0	1.3 ± 0.5	<0.01
• Hot flashes	1.3 ± 0.7	1.5 ± 0.9	1.0 ± 0.0	<0.01
• Increased/decreased appetite	2.2 ± 1.0	2.7 ± 0.8	1.4 ± 0.6	<0.01
• Insomnia	1.2 ± 0.6	1.5 ± 0.7	1.0 ± 0.0	<0.01
• Nausea	1.3 ± 0.6	1.5 ± 0.7	1.1 ± 0.2	<0.01
• Palpitations	1.1 ± 0.3	1.1 ± 0.3	1.0 ± 0.0	0.04
• Swelling in extremities	1.1 ± 0.3	1.1 ± 0.4	1.0 ± 0.0	0.01
**Total physical score (sum of symptoms)**	28.5 ± 6.9	35.0 ± 6.8	22.2 ± 2.3	<0.01
*Affective Symptoms*				
• Angry outbursts	1.8 ± 0.9	2.2 ± 0.9	1.0 ± 0.2	<0.01
• Anxiety/nervousness	1.8 ± 1.0	2.2 ± 1.0	1.1 ± 0.3	<0.01
• Depression	1.5 ± 0.8	2.1 ± 0.9	1.0 ± 0.2	<0.01
• Desire to be alone	1.8 ± 0.9	2.4 ± 0.9	1.1 ± 0.3	<0.01
• Emotional hypersensitivity	2.6 ± 1.0	3.3 ± 0.7	1.4 ± 0.5	<0.01
• Irritability	2.5 ± 1.1	3.3 ± 0.8	1.4 ± 0.5	<0.01
• Mood swings	2.4 ± 1.1	3.0 ± 0.8	1.2 ± 0.4	<0.01
• Tendency to cry easily	2.4 ± 1.2	3.3 ± 0.9	1.2 ± 0.4	<0.01
**Total affective score (sum of symptoms)**	14.7 ± 5.5	20.9 ± 4.7	10.0 ± 1.8	<0.01

**Total symptom score (sum of all symptoms)**	43.1 ± 11.5	55.8 ± 9.7	32.3 ± 3.4	<0.01
*Impact on Activities/Relationships*				
• Relationship discord with family or partner	1.7 ± 0.8	2.2 ± 0.8	1.1 ± 0.3	<0.01
• Relationship discord with friends or coworkers	1.4 ± 0.6	1.7 ± 0.7	1.0 ± 0.2	<0.01
• Poor work performance/attendance	1.4 ± 0.7	1.7 ± 0.8	1.0 ± 0.0	<0.01
• Social isolation	1.8 ± 0.9	2.2 ± 1.0	1.0 ± 0.2	<0.01

*Point values are assigned to each symptom where none = 1; mild = 2; moderate = 3; severe = 4. Symptoms are reported by participants as being experienced most months of the year for at least several days before the start of menstruation;.

^§^P-values from Student’s t-test for continuous variables comparing PMS cases and controls.

We did not find physical activity to be associated with total premenstrual symptom score, either with or without controlling for BMI (Table 3). Each 10 MET increase in activity was associated with a 0.10 point increase in total symptom score in the model adjusted for age, BMI, smoking status, age at first menses, and calcium intake (p = 0.40). Results were similar for affective symptom and physical symptom scores.

**Table 3:** Association between METs* and premenstrual symptom scores, among all participants (n = 414).

	Model 1^§^			Model 2^†^			Model 3^‡^			Model 4^ψ^		
Score	β	SE_β_	p-value	β	SE_β_	p-value	β	SE_β_	p-value	β	SE_β_	p-value
Total symptom score	0.03	0.11	0.76	0.03	0.11	0.80	0.10	0.11	0.40	0.06	0.11	0.62
Affective symptom score	0.05	0.05	0.34	0.05	0.05	0.35	0.07	0.06	0.19	0.07	0.06	0.23
Physical symptom score	-0.02	0.07	0.78	-0.02	0.07	0.74	0.02	0.07	0.73	-0.01	0.07	0.88

*per 10 METs/week;

^§^Adjusted for age;

^†^Adjusted for age and BMI;

^‡^Adjusted for age, BMI, ever smoker, age at first menses, calcium intake;

^ψ^ Adjusted for age, BMI, ever smoker, age at first menses, calcium intake, and reports treating symptoms with exercise.

In analyses comparing women meeting criteria for PMS vs. controls, we found that controls exercised an average of 8.3 METs per week more than cases, although this difference was not significant (54.8 vs. 46.6 METs/week; p = 0.21) (Table 4). After adjusting for BMI and other factors, we did not find physical activity to be related to the prevalence of PMS. Compared to women in the lowest tertile of physical activity, the prevalence odds ratio for women in tertiles 2 and 3 were 1.5 (95% CI: 0.6–3.7) and 0.9 (95% CI: 0.4–2.4), respectively (p-value for trend = 0.85). Similarly, there was no association between METs per week evaluated as a continuous variable and prevalence of PMS (adjusted OR: 1.00; 95% CI: 0.99–1.01).

**Table 4:** Odds ratios and confidence intervals for the association of physical activity and PMS*.

	Cases (n = 80)	Controls (n = 89)	Model 1^§^	Model 2^†^	Model 3^‡^	Model 4^ψ^
	n (%)		OR (95% CI)			
**Physical activity (Median, METs per week)**						
• Tertile 1 (16.3)	24 (30.0)	28 (31.5)	**1.0 (Referent)**	**1.0 (Referent)**	**1.0 (Referent)**	**(Referent)**
• Tertile 2 (39.3)	34 (42.5)	29 (32.6)	1.4 (0.7–2.9)	1.4 (0.7–3.0)	1.5 (0.6–3.7)	(0.6–3.5)
• Tertile 3 (90.5)	22 (27.5)	32 (36.0)	0.8 (0.4–1.7)	0.8 (0.4–1.7)	0.9 (0.4–2.4)	0.9 (0.3–2.2)
	Mean ± SD	Mean ± SD				
**Physical Activity (per 10 METs/week)**	4.7 ± 3.7	5.5 ± 4.4	0.995 (0.988–1.003)	0.995 (0.988–1.003)	0.999 (0.991–1.007)	0.998 (0.990–1.006)

*Among cases and controls (n = 169);

^§^Adjusted for age;

^†^Adjusted for age and BMI;

^‡^Adjusted for age, BMI, ever smoker, age at first menses, calcium intake; Hosmer and Lemeshow Goodness-of-Fit p = 0.8264 for physical activity tertile model, p = 0.9615 for continuous physical activity model;

^ψ^ Adjusted for age, BMI, ever smoker, age at first menses, calcium intake, and reports treating symptoms with exercise.

The null associations between PMS variables and physical activity persisted with further adjustment for use of exercise to treat symptoms (Tables 3 and 4). However, total and physical symptoms scores for PMS were significantly higher in those reporting exercise to treat symptoms (n = 107), with 3 points higher in total symptom score (p = 0.03) and a 2.5 point increase in physical symptom score compared to women not reporting exercise to treat symptoms (p<0.01), after adjusting for physical activity, age, BMI, ever smoker, age at first menses, and calcium intake (Table 5).

**Table 5:** Association of symptom scores with use of exercise to treat menstrual symptoms in participants (n = 414).

	Model Results*		
Score	β	SE_β_	p-value
Total symptom score	2.96	1.32	0.03
Affective symptom score	0.42	0.65	0.52
Physical symptom score	2.54	0.80	<0.01

*Adjusted for physical activity in 10 METs/week, age, BMI, ever smoker, age at first menses, and calcium intake; Note: β values are reported for the difference between women reporting yes to use of exercise to treat symptoms (n = 107) compared to women not reporting use of exercise to treat symptoms (n = 307).

## Discussion

In this cross-sectional study, we found no evidence of an association between physical activity and either prevalent PMS or premenstrual symptom severity. Results were similarly null when we controlled for BMI and when we considered the use of exercise to treat premenstrual symptoms.

We found that many of our participants experienced at least one premenstrual symptom around the time of their expected menses. Identifying modifiable risk factors, such as physical activity, is important to help improve premenstrual symptom severity in women who may not need or want a pharmacological remedy. However, unlike some prior studies [8,11–15,17,18], we did not find a lower prevalence of PMS or premenstrual symptoms in women who participated in high levels of physical activity compared to women who participated in lower levels or no physical activity. We also did not find that a relationship between physical activity and PMS was confounded by BMI. To our knowledge, only one other study of physical activity and PMS adjusted for BMI [21]. This study found an increased risk of PMS and premenstrual symptoms in physically active versus physically inactive women. Additionally, a recent study examining obesity and PMS found that the prevalence of premenstrual symptoms was significantly higher in obese women compared to underweight women (OR = 2.9, 95% CI: 1.1–7.5) [24]. Overall, previous studies have had inconsistent findings, and no association has been found between physical activity and decreased premenstrual symptoms after controlling for BMI, a factor that likely contributes to PMS [23].

Previous studies have hypothesized that frequent physical activity prevents premenstrual symptoms from developing and improves existing monthly symptoms. Some studies have shown that increasing physical activity improves severity of symptoms within 3 months of starting an exercise regimen [16,17,19]. Despite the potential biological connections between physical activity and endorphin levels [5,8], decreased levels of sex hormones [9,10], improvement of oxygen in muscles [9], and a reduction of cortisol levels [9], studies remain largely inconsistent on linking physical activity and reduced premenstrual symptoms. In a study designed similarly to ours, 45.3% of physically active college-aged women had high premenstrual symptom scores compared to 30.6% of college-aged sedentary women based on a self-reported questionnaire on symptom severity [22]. To the contrary, a study that included 748 college-aged women found that the percentage of women experiencing premenstrual and menstrual low back pain, premenstrual and menstrual pelvic pain, premenstrual and menstrual headache, premenstrual nervousness, irritability, anxiety, depression and fatigue were all significantly higher (p<0.05) in women studying in other programs compared to those studying at the Institute of Physical Education. These findings, however, were based on the assumption that physical education students were more active, though this assumption was not validated [15].

We did not exclude women using oral contraceptives from our study for several reasons. Oral contraceptive use was highly prevalent in our study population of young college-aged women. Excluding women using such a common medication would have substantially limited the generalizability of our findings, as well as our statistical power to detect associations. While oral contraceptives are sometimes used to treat menstrual symptoms, we did not find that prevalence of oral contraceptive use varied between PMS cases and controls in our study, or that oral contraceptive use confounded the association between physical activity and either PMS or menstrual symptom severity. Furthermore, in previous population-based studies of PMS conducted by our research team in the Nurses’ Health Study 2 cohort, we have directly assessed whether oral contraceptive use modifies associations between dietary, lifestyle and demographic factors and PMS [34–39]. In all of these studies, we found no difference in results with and without exclusion of oral contraceptive users. Thus, we included oral contraceptive users to maximize our generalizability, power, and consistency with other population-based studies of PMS.

Our study is limited by its cross-sectional study design and cannot explicitly address whether exercise can treat symptoms. The relation of physical activity to symptom experience is complex and multifaceted, and exercise may have benefits for overall well-being among women with menstrual symptoms that are not captured by our analyses. Additionally, we are unable to determine temporality in the physical activity and PMS relationship, where women with symptoms may exercise more to treat their symptoms, as is recommended by the American Congress of Obstetricians and Gynecologists and other groups [40,41]. Temporality bias in our study would bias our results depending on the scenario. Assuming that physical activity is protective, we would expect cases to be less physically active than controls. If cases had higher than expected levels of physical activity, our results would bias toward the null as the exposure levels between cases and controls would be more similar.

Alternatively, if cases become less physically active due to more severe premenstrual symptoms, overall physical activity levels would decrease and bias our results away from the null (making exercise look more protective). This may provide an explanation for previous studies that found an effect. However, we were able to consider whether a participant reported using exercise to help treat premenstrual symptoms, and developed a model to examine reverse causality [42]. This variable did not change the directionality or strength of our findings when added to models as a covariate. However, total and physical symptoms scores were significantly higher in those reporting exercise to treat symptoms, providing evidence that reverse causation is possible.

Strengths of our study include the large range in exercise reported by our participants (0.0–374.3 METs/week), which provided good power to detect an association if one existed. We also used validated PMS and premenstrual symptom questionnaires, which had high sensitivity and specificity for categorizing cases and controls [29,30]. Given the nature of the main study to examine vitamin D status, it is unlikely that we would have been affected by selection bias in this present analysis. Recall bias is also unlikely, as women were asked to report usual symptoms and most of our participants had regular menstrual cycle lengths. Additionally, in our symptom models, we had a broad range of values for all three score variables. Finally, we assessed the impact of many potential confounders, including diet, oral contraceptive use, BMI, menstrual cycle characteristics, and smoking history.

## Conclusions

In conclusion, our results do not support an inverse physiologic relation between physical activity and the prevalence of PMS or the severity of premenstrual symptoms. Instead, our results suggest that women experiencing a greater number of physical premenstrual symptoms are more likely to exercise than women reporting fewer premenstrual symptoms. Ours is among the first studies to directly consider whether the suggested benefits of exercise for treating PMS may be attributable to reverse causation. These findings contribute further evidence that increasing physical activity may not be effective for treating moderate to severe PMS. Instead, women experiencing significant PMS may receive greater benefit from following clinical recommendations supported by more evidence, such as dietary modifications and medication use.

## Supporting Information

S1 Appendix Supplemental Information on the Definition of PMS Cases and Controls. (DOCX)

## Abbreviations

BMI: Body mass index
CI: Confidence interval
COPE: Calendar of Premenstrual Experiences
METs: Metabolic equivalent task-hours
OR: Odds ratio
PMS: Premenstrual syndrome
SD: Standard deviation
SE: Standard error
