# Supplementary material for: Recreational Physical Activity and Premenstrual Syndrome in Young Adult Women: A Cross-Sectional Study
Source: PLoS One. 2017 Jan 12;12(1):e0169728. doi: 10.1371/journal.pone.0169728 (PMC5231278; doi:10.1371/journal.pone.0169728)
Supplement: S1 Appendix — (DOCX) [file pone.0169728.s001.docx]

**S1 Appendix: Supplemental Information on the Definition of PMS Cases and Controls**

A modified version of the Calendar of Premenstrual Experiences designed by Mortola *et al*. [1] was used to collect information on premenstrual symptoms and to define cases and controls in our study. Participants were asked if they experienced 26 different symptoms (Table S1) “most months of the year, for at least several days before your menstrual period begins,” and to indicate the usual severity of each symptom as none, mild, moderate, or severe. Participants were asked not to include symptoms that they experienced throughout their entire menstrual cycle, or symptoms that started only when their period started. For each symptom, we assigned point values as follows: none = 1; mild = 2; moderate = 3; severe = 4. We then summed across all symptoms to derive a total premenstrual symptom score (range of possible scores = 26 – 104). We also calculated subscores for affective symptoms (n = 8 symptoms; range of possible scores = 8 – 32) and physical or behavioral symptoms (n = 18 symptoms; range of possible scores = 18 – 72).

| **S1 Table: Premenstrual symptoms included in data collection (n=26)** |
| --- |
| *Physical Symptoms (n=18)* |
| Abdominal bloating |
| Abdominal cramping |
| Acne |
| Back pain |
| Breast tenderness |
| Confusion |
| Diarrhea/constipation |
| Dizziness |
| Fatigue |
| Food cravings |
| Forgetfulness |
| Headache |
| Hot flashes |
| Increased/decreased appetite |
| Insomnia |
| Nausea |
| Palpitations |
| Swelling in extremities |
| *Affective Symptoms (n=8)* |
| Angry outbursts |
| Anxiety/nervousness |
| Depression |
| Desire to be alone |
| Emotional hypersensitivity |
| Irritability |
| Mood swings |
| Tendency to cry easily |

Participants were asked to indicate whether their overall severity of premenstrual symptoms was “minimal” (no effect on normal activities); “mild” (noticeable, but not troublesome); “moderate” (interferes with normal activities); or “severe” (intolerable, prevents normal activities). They were also asked to report the number of days before their period when symptoms usually began, and the number of days symptoms lasted after their period began. Women were asked to report whether they experienced relationship discord with family or a partner; relationship discord with friends or coworkers; poor work performance or attendance; or social isolation, and to indicate the severity of each as not a problem, mild, moderate, or severe. Women were also asked to report whether they had ever been clinically diagnosed with PMS, and whether they used exercise, dietary supplements or other factors to treat their symptoms.

To determine whether women were experiencing other psychiatric conditions similar to PMS, we asked all participants about history of depression and bipolar disorder and current use of anti-depressant medications. In 2008, we added the General Depression 20-item Sub-scale of the Inventory of Depression and Anxiety Symptoms (IDAS) to our study [2], which was completed by 175 participants.

Responses to the premenstrual symptom questionnaire were also used to identify women meeting clinical criteria for PMS [3,4]. **Cases** were defined as women who reported:

1. at least one physical symptom rated as “moderate” or “severe”
2. at least one affective premenstrual symptom rated as “moderate” or “severe”
3. overall symptom severity or impact of symptoms on life activities and relationships as “moderate” or “severe
4. symptoms beginning within 14 days of the start of menses
5. symptoms ending within 7 days of the start of menses
6. symptoms are absent in the mid to late follicular phase
7. no evidence of a comorbid psychiatric disorder (e.g., history of depression or bipolar disorder; current anti-depressant use; IDAS score ≥50)

**Controls** were defined as women who reported:

1. overall symptom severity of “none”, “minimal” or “mild”
2. impact of symptoms on life activities and relationships of “not a problem” or “mild” for all items
3. no affective symptom rated as “moderate” or “severe”
4. no previous clinical diagnosis of PMS
5. no evidence of a comorbid psychiatric disorder (e.g., history of depression or bipolar disorder; IDAS score ≥40)

References

1. Mortola J, Girton L, Beck L, Yen S. Diagnosis of premenstrual syndrome by a simple, prospective, and reliable instrument: The Calendar of Premenstrual Experiences. Obstet Gynecol. 1990;76: 302–307. Available: http://journals.lww.com/greenjournal/abstract/1990/08000/diagnosis_of_premenstrual_syndrome_by_a_simple,.31.aspx

2. Watson D, O’Hara MW, Simms LJ, Kotov R, Chmielewski M, McDade-Montez EA, et al. Development and validation of the Inventory of Depression and Anxiety Symptoms (IDAS). Psychol Assess. 2007;19: 253–268. doi:10.1037/1040-3590.19.3.253

3. Bertone-Johnson ER, Ronnenberg AG, Houghton SC, Nobles C, Zagarins SE, Takashima-Uebelhoer B, et al. Association of inflammation markers with menstrual symptom severity and premenstrual syndrome in young women. Hum Reprod. 2014;0: 1–8. doi:10.1093/humrep/deu170

4. Bertone-johnson ER, Houghton SC, Whitcomb BW. Association of Premenstrual Syndrome with Blood Pressure in Young Adult Women. 2016;0: 1–7. doi:10.1089/jwh.2015.5636
